# Acknowledgment

**Published:** 1866-05

**Authors:** 


### Editorial.

### ACKNOWLEDGMENT.

Some three months ago, the Ohio College of Dental Surgery received of Dr. A. Preterre, Paris, France, a liberal contribution in the form of six beautiful, interesting and instructive preparations, exhibiting various deformities of the mouth and continguous parts, together with the methods and appliances for remedying the same; the latter of which exhibited a great degree of ability and tact, in that direction. They were examined by, and elicited much admiration and interest, from the members of the Dental College Association, recently in session. The preparations were each explained by Dr. McKellops, of St. Louis, recently of Paris, who had enjoyed the privilege of Dr. P.'s personal explanations, and saw some of the appliances in actual use in the patient's mouths. We here give Dr. P.'s written description of the different pieces:

The 1st is a head, representing the loss of the right ascending branch of the lower Maxillary, with two artificial pieces remedying the defect. The upper piece is intended to oppose itself to the approximation of the superior Maxillary, when any part of the lower Maxillary is deficient.

The 2nd represents the loss of one of the superior Maxilla, with rigid palate; the wearer of this fixture has obtained a most perfect articulation of sounds, smokes, eats and drinks, as well as any one. The pieces has now been in use ten years. The inspection of this piece must modify, considerably, the opinions expressed on the utility and absolute necessity of a flexible velum, since a peıfect articulation is obtained by a rigid palate.

The 3rd represents the loss of a portion of the nerve, and the piece repairing the loss.

The 4th represents the deviation or approximation of the upper jaw when the lower Maxillary is absent. It is to overcome this difficulty that we have invented the apparatus above spoken of. The cast was taken from an old soldier.

The 5th is a case in which the entire tongue was removed by an operation. It is remarkable, that the absence of the tongue produces the same deficiency in pronunciation as the loss or absence of the palate. The appliance, or artificial tongue, gave very satisfactory results.

The 6th represents a most complicated case of cleft palate, with about three fourths of an inch anterior projection of the central incisors, with their alveolus. This projection was removed entire, and an artificial palate, with teeth, applied; and after proper training, the issue was very satisfactory.

There is material in these preparations for study and investigation.

The thanks of the College, and all interested in it, is certainly due to Dr. Preterre for this valuable presentation, and also to Dr. McKellops, for the effort which he exercised in giving this contribution its direction thitherward, and for the interesting manner in which they were explained by him to the Association.

### NASHVILLE DENTAL SOCIETY.

The dentists of Nashville and vicinity, organized a society in October last. The meetings are held on the second Tuesday of each month. All its members are wide awake men, and will produce good results in this effort.

The officers are, Dr. W. H. Morgan, Pres't; Dr. S. J. Cobb, Vice-Pres't; Dr. J. C. Ross, Recording Secretary; Dr. R. Russell, Corresponding Secretary.

It is very desirable that all over our country local societies be formed, at the earliest possible period.

